# The place of knowledge management in influencing lasting health change in Africa: an analysis of AMREF's progress

**Published:** 2012-12-25

**Authors:** Rumishael Shoo, Willy Matuku, Jane Ireri, Josephat Nyagero, Patrick Gatonga

**Affiliations:** 1 African Medical and Research Foundation, Nairobi, Kenya

**Keywords:** Knowledge management, databases, learning culture, Africa

## Abstract

**Introduction:**

AMREF (African Medical and Research Foundation) developed a Knowledge Management Strategy that focused on creating, capturing and applying health knowledge to close the gap between communities and health systems in Africa. There was need to identify AMREF's current Knowledge Management implementation status, problems and constraints encountered after two years of enforcement of the strategy and suggest the way forward.

**Methods:**

This study was conducted between October 2011 and February 2012. Quantitative data on number and foci of AMREF research publications were collected using a questionnaire. Focus group discussions and in-depth interviews were used to gather data on explanations for the trend of publications and the status of the implementation of the 2010-2014 Knowledge Management Strategy. Quantitative data was analysed using SPSS computer software whereas content analysis of themes was employed on qualitative data.

**Results:**

Between 1960 and 2011, AMREF produced 257 peer reviewed publications, 158 books and manuals and about 1,188 technical publications including evaluations, guidelines and technical reports. However, the numbers of publications declined from around the year 2000. Large quantities of unpublished and unclassified materials are also in the custody of Heritage. Barriers to Knowledge Management included: lack of incentives for documentation and dissemination; limited documentation and use of good practices in programming; and superficial attention to results or use of evidence.

**Conclusion:**

Alternative ways of reorganizing Knowledge Management will enable AMREF to use evidence-based knowledge to advocate for appropriate changes in African health policies and practices.

## Introduction

*“Knowledge is power; making our knowledge widely and readily available will empower others to come up with solutions to the world's toughest problems.”* World Bank Group President Robert B. Zoëllick, April 2012 [1]

Knowledge management (KM) has been defined in the African Medical and Research Foundation (AMREF) strategy as the process by which the organization creates, captures, stores and applies health knowledge to support and close the gap between communities and their health systems in Africa [2]. The strategy states the vision of AMREF as – being the leading source of Africa's health knowledge, working with partners to generate, store, share and apply knowledge to ensure better health for Africa. The organization's business plan further states the AMREF vision as spearheading lasting health change in African communities [3].

A review of AMREF performance in generating and sharing knowledge that it creates was undertaken by the Joint CIDA/Sida review mission of 2006 [4]. The review found a declining trend in research and publications at AMREF and the need for the organization to address both capacity and organizational commitment to research. AMREF took deliberate action to collect documents and articles published from country offices after this review. A number of positive steps were also undertaken by the organization including the establishment of a Health Systems Policy and Research Directorate, development of a Knowledge Management Strategy and a Research Strategy. The situation improved slightly but was not sustained. A KM core group was established as part of the Knowledge Management Strategy implementation. The core group draws individuals from across the organization. We set out to investigate the extent to which the AMREF vision and Knowledge Management strategy are being realized with the following objectives: to study the focus and trend of AMREF research outputs since its establishment; to analyze the knowledge storage and dissemination capacity; to elucidate constraints for knowledge management in AMREF; and to gather suggestions and recommendations on how to improve knowledge management in the organization.

## Methods

A questionnaire was developed to capture data on research undertaken by all the seven AMREF country offices in Africa except Senegal whose office opened recently. We also used the resource centre database which is a central AMREF repository to collect data on numbers of AMREF publications from 1960 to 2011. By nature of their activities, the 12 countries in Europe and North America were excluded. To analyze constraints to the conduct of research, we conducted focused group discussions and in-depth interviews in Tanzania, Uganda, Ethiopia and Kenya AMREF country offices. In-depth interviews with managers of programmes to get explanations for the observed trends in knowledge generation were done. Focus group discussions were held with key departments and directorates to identify Knowledge Management capacity and constraints to research and to seek possible solutions.

## Results

The findings have been addressed in various themes including the focus and trend of AMREF research outputs, Storage and Dissemination Capacity, Constraints for Knowledge Management in AMREF, and suggestions for improving Knowledge management.

### Focus and Trend of AMREF Research Outputs

**Figure 1:**
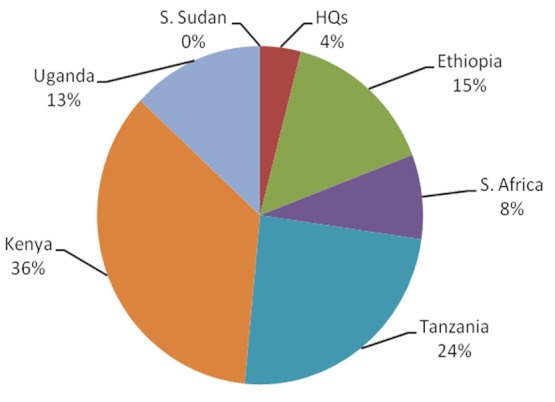
Distribution of AMREF (African Medical and Research Foundation) research projects by country (n=61)

**Figure 2:**
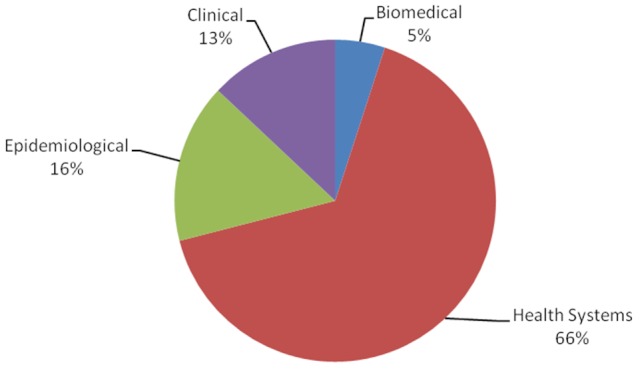
Categorisation of AMREF (African Medical and Research Foundation) research projects by type (n=61)

The number of research projects undertaken or completed by September 2011 by AMREF staff was 61. The distribution by country and type is shown on Figure 1 and Figure 2. An analysis in March 2012 based on documentation and peer reviewed materials held by the AMREF Resource Centre found that between 1960 and 2011, AMREF produced 257 peer reviewed publications, 158 books and manuals and about 1,188 technical publications including evaluations, guidelines and other technical reports. The decline of peer reviewed articles started in the year 2000 but there has been a rapid increase of technical documents afterwards. The latter also started declining from around 2005. Production of books and monographs however peaked at around 2009 but also started declining thereafter.

There were several explanations given by long serving AMREF staff for the declining trend. The commendable increase in the number of technical publications was said to be partly due to involvement of the Masters and Diploma students trained by AMREF in research. The decline in peer reviewed publications was attributed by staff to the limited time and institutional support to research. Some staff felt that as long as research is neither recognised nor rewarded by AMREF, the declining trend will continue.

*“Research is not specified as a key activity in our job description. Therefore it is not included in the annual work plans and those who engage in research activities do so in their free time in the night and/or weekends”* - an AMREF employee

The decline in books and manuals was explained by staff as largely due to Health Learning Materials Programme switching more to e-learning. Essentially the production of AMREF Health Learning Materials Programme has scaled down in recent years.

AMREF instituted Annual Technical Review Meetings in countries and a Biennial Programme Review Meeting organization-wide. The main agenda of these meetings is sharing and reviewing programme experiences, lessons learnt and research output. In 2011, a total of 80 abstracts were submitted and presented by staff in Annual Technical Review meetings in Tanzania, Kenya and Ethiopia alone. In April 2012, the first Biennial Programme Meeting brought together 145 participants from 7 countries in Africa and 8 in Europe and North America. A total of 114 high quality abstracts were received and reviewed using an agreed criteria. Eighteen oral presentations on AMREF systems and policies and another 70 oral scientific presentations and 19 good quality posters on AMREF programmatic issues were made. AMREF has provided support for development of full papers of these presentations for publication in the Pan African Medical Journal (PAMJ) as a special supplement.

### Storage and Dissemination Capacity

AMREF has in existence an elaborate central and web-based system (Intranet) for storage, transfer and retrieval of its documents. Access to this system relies on an AMREF Wide Area Network that at the moment covers offices in Africa only. Access and retrieval from this system has been good and the products are easy to use. All Key AMREF documents are supposed to be stored here. The system is fully web-based and is accessible from anywhere and at any time. It has capability to provide discussion forums, search, host databases and work offline among others. It is a centralised system with distributed access. Upload of contents is centralised. Some staffs are unaware of its capability.

AMREF has put in place a video conference facility at its headquarters and plans to provide similar facilities to its others offices. This will allow anyone to communicate with others from any place at any time. This infrastructure supports communication and information sharing and virtual teams. In addition staffs use other virtual systems like Skype, go-to-meeting etc. However, internet downtimes have been a challenge to users outside the headquarters in Nairobi. Interviewed staff suggested that AMREF should continuously build staff skills in intranet usage. It should also scan the market regularly for improved internet possibilities in order to improve connectivity across all its offices. Additionally, an internet backup should be put in place.

*“Not many of us are fully aware of capabilities of the intranet. The organization should build our capacity so that we can exploit the full potential of some of these powerful knowledge management tools”* – an AMREF employee

AMREF has established reputable Online Knowledge sharing platforms which encourage sharing across the Foundation while at the same time reaching stakeholders with the right information at the right time. These platforms include: AMREF Website and Intranet; Digital Library; ART Knowledge Hub Online Platform; ART Online Discussion forum; various e-bulletins which include *Gumzo e-bulletin, AMREF Library e-bulletin, ART Knowledge Hub e-Bulletin*; Maternal Newborn and Child Health Online platform and the AMREF Heritage Stand-alone database.

Apart from the above platforms, AMREF has accumulated large amounts of documents which still mainly remain in form of donor reports, surveys and evaluation reports. Only a small amount of this (grey) literature is captured, processed, and shared using the corporate knowledge and information dissemination platforms such as the main AMREF Website, the Intranet and the Digital Resource Centre for example. In addition, a small amount of such documentation has been published into Health Learning Materials and in peer reviewed journals. Where some AMREF staff have published in peered reviewed journals, the study showed that a number of these publications were not in the Resource Centre database.

### Constraints for Knowledge Management in AMREF

At the Resource Centre databases it was observed that systems which have been developed to enable internal and external sharing of information and knowledge remain under-utilised. The buy-in of Knowledge Management by staff and the understanding of the KM system remain also low. Staffs do not share widely prepared documents as they are perceived as simply not important for sharing. Some Country Offices do not have libraries or resource centres to act as repositories to access the latest information and increase knowledge. Directorates and Departments continue to manage their own technical databases with limited sharing even across departments. The good news is that the trend is now changing and directorates are centralising the databases. Equally, access to Intranet as well as to these databases has improved and more staffs are visiting the Intranet.

AMREF over the years has mainly published its Health Learning Materials in English language therefore leaving out other key languages such as French and Portuguese. This has created a communication barrier with other African countries where the official language is not English.

*“AMREF is now operating in more countries than before; including in French speaking and Portuguese-speaking countries. For our learning materials to be useful in all these countries, the materials will have to be translated into more languages.”* – an AMREF employee

It is recognised that to some extent that knowledge sharing takes place in AMREF but in an unstructured way. Most of this sharing is unconscious, limited and inefficient. In its over 50 years of existence, AMREF has gathered a lot of information and knowledge through project activities, many of which comprise best practices and lessons learnt. However, there has been the general inability to transfer best practices and lessons learnt from one part of the organization to another, resulting in re-invention.

Other common barriers to knowledge sharing identified include staff tending to focus on their own team or business units and seeing no responsibility beyond their core business boundaries. They rarely seek for alternative solutions for problems beyond the solutions they have used in the past thus staying within their comfort zones. Employees perceive that making mistakes is wrong and therefore are not keen for honest and open sharing of their experiences especially on unsuccessful projects.

*“We do not have a well-developed culture of knowledge sharing. Some staff think that seeking knowledge from others is a sign of weakness on your part.”* – an AMREF employee

Inadequate awareness of Knowledge Management and its potential impact on improved performance is also a problem as is lack of a Management Information System (MIS) that informs the various institutional documents produced annually. Staff are usually engaged in vertical reporting activities and do not have time to learn and share from experience. Knowledge Management is not adequately integrated into staff roles. In some cases ddocumentation is lengthy and tedious to both write and read. Knowledge-based activities are often seen as additional work that can be done when staffs are “not busy”. Knowledge management has not been recognised during programming and development of job description and followed up during implementation and appraisals. There are limited strong institutional communities of practice in place at AMREF to share both tacit and explicit knowledge.

### Suggestions for improving Knowledge management

The current problems may be solved if staffs understand they have roles to play beyond their business units. Development and implementation of organisation wide change management programmes is also necessary as is nurturing of performance cultures based around teamwork. Senior Management should be more involved and informed on knowledge management. The organization should continue awareness creation programmes to cover all offices.

*“We all need to be made aware of the importance and practice of knowledge management; across all staff cadres”* – an AMREF employee

It is necessary to develop a Management Information System in order to capture key documents, products and store them in the central depository (Intranet). Development of documentation skills across the organization is crucial. Lessons learned should be fed back into the organization so that improvements can be made. Each project should identify someone responsible and accountable for KM. Strategies for capturing tacit knowledge should be improved. Lastly, KM should be fully integrated into staff roles.

*“Knowledge management should be part of staff's job description. That way there will be a system of accountability for creation, storage and dissemination of knowledge”* - an AMREF employee

### Recommendations on Storage and Dissemination

There needs to be a clear distinction between technical information and non-technical information and clear identification of where the knowledge, its repackaging and dissemination of such information should be addressed. Investing in new systems may not be the right solution. AMREF should utilise systems already available and ensure their full potential is exploited.

*“We always talk of investing in newer technologically advanced systems. It is time that we now concentrated on fully making use of what is already available.”* – an AMREF employee

There should be clear distinction on what information AMREF should share externally and what should be limited for access within AMREF. Proposals and external project reviews for example, should be shared with restriction. Such information should be shared internally through the central Intranet. KM champions should work further to ensure buy-in by all staff. Such championship should be across the organization. The AMREF Intranet still remains the main internal communication channel for all staff in AMREF. Though training has been done especially on full utilisation of the Intranet, further training needs to be done to ensure that the system is well utilised for internal communication. There is need to develop practical non monetary incentives to staff who document and share their knowledge. All available information platforms should be accessible by all. This should stop the culture of asking for information and forwarding such documentation via personal communication channels.

## Discussion

The ultimate aim of the existing Knowledge Management and the recently developed Research Strategy in AMREF is to set up a culture of knowledge generation for influence of policy and practice. This is achievable with the establishment of a strong Knowledge management system. Through this, the organization can exploit the potential of creating internal and external communities of practice. In fact, Guptin identifies five areas that need to be addressed in setting up an efficient Knowledge Management system in health care [5]. These are communities of practice, content management, knowledge and capability transfer, performance results tracking and technology and support infrastructure. According to Wenger, communities of practice are “groups of people who share a concern or a passion for something they do and learn how to do it better as they interact regularly” [6]. These individuals interact regularly in order to share experiences in problem solving, seeking new ways of doing things, seeking new knowledge like preserving assets, discussing developments, documentation of practices, visits and mapping out knowledge and identifying their knowledge gaps. AMREF has been working on a programme to ensure it becomes a hub for exchanging health knowledge throughout Africa. It moved from doing this in the traditional meetings and workshops and now hosts a number on Networks ranging from Anti - retroviral treatment, Reproductive and Child Health and lately hosting a network in Collaboration with USAID's Strengthening Health Outcomes through the Private Sector (SHOPS) project.

Capacity for generating knowledge exists within AMREF. On content management, AMREF hosts a team of highly qualified technical group in charge of the technical programmes in Reproductive and Child Health (RCH), Malaria, HIV/AIDS and TB, Water and Sanitation, Clinical and Diagnostics Services and Research. These technical personnel generate and update knowledge content in collaboration with country offices. They check the quality and accuracy of information. The communication and advocacy group packages and market the content for policy influence.

AMREF has developed a robust Monitoring and Evaluation System as part of Programme Management Unit. Results Based Frameworks have been developed to monitor the outputs of all these programmes. What needs to be developed further is a system to ensure that tested results, experiences and lessons learnt from these programmes are well documented and disseminated for global consumption.

A good Knowledge Management system provides the right information to the right person at the right time with the aim of attaining greater competitive advantage [7]. This is what AMREF aspires to achieve. AMREF will move beyond sharing explicit knowledge through publishing of research to establishing systems within the organization of sharing tacit knowledge through attachments, mentoring systems and good succession planning. Strengthening of monitoring systems to ensure long term impacts through longitudinal studies is part of the research strategy.

A number of reasons that hinder development of a knowledge sharing culture to prevent knowledge loss and gain competiveness have been identified at AMREF. Chan and Chan [8] have identified a number of factors that facilitate strong KM culture. These include an organizational culture and structure that promotes KM [9]. Management support and especially the executive management are crucial if KM is to succeed. This includes the identification and recognition of KM champions [10]. In the case of AMREF, this issue needs further commitment. Although AMREF has a good KM strategy, this strategy has not been widely disseminated and internalized within the organization. The KM champions have to include the most influential people in the organization. AMREF has developed a business plan and a research agenda that includes a KM component linked to the plan. As part of the research strategy, an incentive system is to be implemented for nurturing a research culture within the organization. Integration of monetary and non monetary systems is crucial for success [11].

A number of factors were identified by the resource centre and IT units in AMREF as to why staff do not share or fully utilize available resources. It is important that staff are trained to use the systems available especially if they are technology based [12]. Staff should participate and contribute to the design of the system for them to own it. A top down approach to design or lack of trust in the management also tends to affect KM [13].

Lastly, it should be emphasized that it takes time and money to develop a good KM system. Investment in an efficient KM system is not only a great motivation for staff but a very cost-effective investment. Nothing is more frustrating like a poorly managed system that frequently malfunctions or is overloaded with irrelevant content. These can be great impediments [14]. However, the good use of the KM system is the most important thing in an organization.

## Conclusion

AMREF remains a leading knowledge hub in health for Africa. The organization has an impressive record of research and publications but its systems for managing and sharing this knowledge to influence policy and practice in Africa need urgent improvements to realize this potential.
